# Insulin resistance as a mediator of the association between obesity, high-intensity binge drinking, and liver enzyme abnormalities in young and middle-aged adults: a cross-sectional study

**DOI:** 10.3389/fnut.2025.1554392

**Published:** 2025-07-24

**Authors:** Jiayi Zhu, Yinglong Duan, Ying Li, Yi Zhou, Zitong Lu, Nandan Chen, Juan Luo, Xingxing Wang, Xiaoqian Dong, Andy S. K. Cheng, Yuan Liu

**Affiliations:** ^1^Health Management Medicine Center, The Third Xiangya Hospital, Central South University, Changsha, China; ^2^Nursing Department, The Third Xiangya Hospital, Central South University, Changsha, China; ^3^Xiangya Nursing School, Central South University, Changsha, China; ^4^School of Health Sciences, Western Sydney University, Sydney, NSW, Australia; ^5^Department of Anesthesiology, The Second Xiangya Hospital, Central South University, Changsha, China

**Keywords:** insulin resistance, obesity, binge drinking, liver enzymes, metabolic dysfunction, young and middle-aged

## Abstract

**Background:**

Binge drinking (BD) and obesity are well-established risk factors for liver enzyme abnormalities, but how varying intensities of BD interact with obesity to affect liver function remains unclear. This study aims to examine whether insulin resistance (IR) mediates the associations between different levels of BD, obesity, their interaction, and liver enzyme abnormalities.

**Methods:**

This cross-sectional study included 137,878 young and middle-aged adults who underwent physical examinations in southern China between August 2017 and March 2024. BD was self-reported, and IR was assessed using the triglyceride–glucose (TyG) index. Causal mediation analysis within the counterfactual framework was used to quantify the mediating role of the TyG index in the associations involving BD intensity, obesity, their interaction, and liver enzyme abnormalities.

**Results:**

The interaction between obesity and high-intensity binge drinking (HIBD) was significantly associated with liver enzyme abnormalities (OR, 1.591; 95% CI, 1.401–1.806). IR, measured by the TyG index, statistically accounted for 36.6% (OR, 1.034; 95% CI, 1.029–1.039) of this association, exceeding the proportion explained in the HIBD alone (25.9%) or obesity alone (16.7%) pathways. No significant mediating effect of IR was observed for non-BD or low-intensity BD, regardless of obesity status.

**Conclusion:**

The TyG index serves as a critical mediator in the synergistic effects of HIBD and obesity on liver enzyme abnormalities. Targeting IR and reducing the intensity of alcohol consumption may help mitigate liver injury in young and middle-aged adults with obesity.

## 1 Introduction

Abnormal liver enzymes are key indicators of hepatic injury (1) and are closely associated with chronic liver and metabolic diseases (2). In recent years, the prevalence of liver enzyme abnormalities has increased among young and middle-aged populations (3), largely driven by increasing rates of obesity (4) and binge drinking (BD) (5). Liver health in this demographic not only affects the individual quality of life but also has broader socioeconomic implications. Therefore, understanding the underlying mechanisms of liver enzyme abnormalities in this population holds significant clinical and public health importance.

Young and middle-aged adults exhibit the highest prevalence of BD, with an increasing trend observed in even younger age groups (6, 7). BD is a major behavioral risk factor for liver damage, and its intensity has a substantial impact on metabolic health (8). High-intensity binge drinking (HIBD) causes hepatocellular injury through toxic metabolites such as acetaldehyde and reactive oxygen species (ROS) (9), including ROS generated via neutrophil cytosolic factor 1-dependent pathways (10) and gastrin-releasing peptide receptor-mediated activation of NADPH oxidase 2 (11). These ROS suppress AMP-activated protein kinase and the anti-inflammatory microRNA-223 (10, 11), promote hepatic lipid accumulation and inflammation, disrupt mitochondrial function, and trigger oxidative stress-induced hepatocyte death (12–14), ultimately elevating the risk of liver enzyme abnormalities. Obesity, another global public health concern (15, 16), contributes to liver injury through chronic low-grade inflammation and oxidative stress driven by excess adipose tissue accumulation (17–19). Moreover, obesity increases hepatic susceptibility to alcohol-induced damage, suggesting a synergistic interaction that aggravates liver injury beyond the independent effects of either condition (20–22). However, the biological mechanisms underlying these interactions, particularly the synergistic effects of obesity and BD on liver injury, remain insufficiently understood and warrant further investigation (23, 24).

Insulin resistance (IR), a hallmark of metabolic disorders, may serve as a shared mediating mechanism and a central contributor to the synergistic effects of BD and obesity on liver injury in young and middle-aged populations. Obesity-induced chronic inflammation and lipid accumulation impair insulin signaling pathways by promoting the secretion of pro-inflammatory cytokines such as tumor necrosis factor-α (TNF-α), interleukin-6 (IL-6), and interleukin-12 (IL-12), as well as by increasing the release of free fatty acids (FFAs) from visceral adipose tissue (VAT), thereby reducing insulin sensitivity and aggravating intrahepatic lipid deposition and oxidative stress (25, 26). Simultaneously, BD impairs insulin signaling via acetaldehyde and ROS, which inhibit the phosphorylation of insulin receptor substrate-1 (IRS-1) and reduce glucose transport efficiency (27, 28). Experimental studies further indicate that HIBD exacerbates IR in individuals with obesity, intensifying metabolic dysfunction (28). IR contributes to liver injury through multiple interrelated mechanisms, including increased hepatic *de novo* lipogenesis, inadequate suppression of gluconeogenesis, elevated FFA influx from adipose tissue, and intracellular accumulation of lipotoxic intermediates such as diacylglycerol and ceramides (29, 30). These metabolic disturbances promote mitochondrial dysfunction, oxidative stress, and activation of inflammatory pathways, ultimately leading to hepatic steatosis, inflammation, and fibrosis (29–32). Within this context, IR may not only magnify the independent effects of obesity and BD on liver dysfunction but also serve as a key mediator of their synergistic impact on liver enzyme abnormalities (31, 32).

Given these considerations, this study hypothesizes that IR mediates the associations between BD, obesity, and their interaction with liver enzyme abnormalities. The magnitude of this mediating effect is expected to vary by BD intensity and to be the strongest in the interaction between HIBD and obesity. Utilizing data from a large cross-sectional health survey in China, this study aims to examine these mediation effects across different levels of BD in young and middle-aged adults and to quantify the extent of the mediating effects.

## 2 Methods

### 2.1 Participants

Participants in this cross-sectional study were recruited through convenience sampling from the Health Management Center of a comprehensive hospital in China. The inclusion criteria were as follows: (1) age between 18 and 59 years; (2) willingness to participate free of charge; and (3) sufficient reading comprehension to complete a self-reported health questionnaire in Chinese. The exclusion criteria included (1) diagnosis of a severe mental disorder and (2) diagnosis of some chronic conditions such as hypertension, stroke, coronary heart disease, chronic kidney disease, chronic gastritis or peptic ulcer, chronic obstructive pulmonary disease, chronic pancreatitis, chronic hepatitis or liver cirrhosis, hyperuricemia, or malignancy, as well as the current use of medications for these conditions.

### 2.2 Study design and procedures

This single-center cross-sectional study was conducted from August 2017 to March 2024. The study followed the Strengthening the Reporting of Observational Studies in Epidemiology (STROBE) checklist to ensure comprehensive and transparent reporting. Based on the hypothesized framework, the primary exposures included five levels of BD intensity, obesity, and their interaction. The proposed mediator was IR, assessed using the triglyceride–glucose (TyG) index, and the primary outcome was liver enzyme abnormality, defined by serum alanine aminotransferase (ALT) and aspartate aminotransferase (AST) levels.

Before undergoing the health examination, all participants received a text message with a link to an electronic health self-assessment questionnaire, which they completed online. After the examination, laboratory test results were extracted from the hospital’s electronic medical record system by the research team. Written informed consent was obtained from all participants. Participation was entirely voluntary, and no financial compensation was provided. This study protocol was approved by the Ethics Committee of the Third Xiangya Hospital, Central South University (NO. quick-24556). A total of 137,878 participants were included in the final analyses, providing sufficient statistical power to detect both main and interaction effects, as well as the mediation pathways.

### 2.3 Data collection

General information was collected from participants via a health self-assessment questionnaire designed by researchers, including sex, age, alcohol consumption, smoking status, and exercise. Venous blood samples were collected from participants after an 8–12-h fasting period, and trained medical technicians measured serum triglycerides (TG), total cholesterol (TC), high-density lipoprotein cholesterol (HDL-c), low-density lipoprotein cholesterol (LDL-c), and fasting blood glucose (FBG) using standardized laboratory methods. Variables with a *p-*value of < 0.05 in univariate analyses and those identified as potential confounders based on prior literature were included in the multivariable model. Detailed measurement methods for covariates are provided in the Supplementary materials.

#### 2.3.1 Obesity assessment

The height and weight of participants were measured by trained medical staff using a calibrated electronic stadiometer, with height recorded to the nearest 0.1 cm and weight recorded to the nearest 0.1 kg. Body mass index (BMI) was calculated as the ratio of weight (kg) to height squared (m^2^). According to the World Health Organization criteria for Asian adults, BMI was categorized as follows: underweight (< 18.50 kg/m^2^), normal weight (18.50–22.90 kg/m^2^), overweight (23.00–24.90 kg/m^2^), and obesity (≥ 25.00 kg/m^2^) (33). In this study, the underweight group was merged with the normal-weight category due to the small number of participants in the underweight group.

#### 2.3.2 Insulin resistance assessment

IR in this study was assessed using the TyG index, which has been validated as a reliable surrogate marker for IR. Compared to the hyperinsulinemic-euglycemic clamp technique, the TyG index is more practical and suitable for large-scale epidemiological studies (21, 34). The TyG index was calculated as ln[fasting TG (mg/dl) * FBG (mg/dl)/2]. Serum TG levels were measured using the triglyceride lipase method, and FBG was assessed using the glucose oxidase method. Both tests were conducted using an automated biochemical analyzer.

#### 2.3.3 Definition of liver enzyme abnormalities

In this study, liver enzyme abnormalities were defined based on the ALT and AST levels, which are commonly used markers of hepatocellular injury. Participants were considered to have abnormal liver enzymes if they met any of the following sex-specific criteria: ALT > 50 U/L for men, ALT > 35 U/L for women, or AST > 34 U/L. (16, 35). The ALT and AST levels were measured using the enzymatic rate method on an automated biochemical analyzer.

#### 2.3.4 Evaluation of binge drinking

Information on alcohol consumption was collected through the health self-assessment questionnaire, including drinking frequency per week, volume per drinking occasion, and type of alcoholic beverage. The questionnaire was developed based on previously published surveys assessing drinking patterns in epidemiological studies (36, 37). To enhance cultural relevance and comprehension, the instrument was adapted linguistically (e.g., using the traditional Chinese unit “liang” instead of milliliters). The questionnaire demonstrated high response completeness, with a low missing data rate of 0.72% for BD variables. Based on alcohol concentrations—53% for Chinese liquor, 12% for wine, and 4% for beer—and an alcohol density of 0.79 g/mL (38), the standard alcohol content per milliliter was calculated as follows: Chinese liquor, 0.53 * 0.79 = 0.42 g/mL; wine, 0.12 * 0.79 = 0.09 g/mL; beer, 0.04 * 0.79 = 0.03 g/mL. The total amount of alcohol consumed per occasion was calculated by multiplying the standard alcohol content by the volume consumed. In this study, BD behavior was categorized based on the amount of alcohol consumed per drinking occasion. Non-BD was classified as an alcohol intake of ≤ 60 g per occasion for men and ≤ 40 g for women. Level I BD was defined as intake > 60 g but ≤ 120 g for men and > 40 g but ≤ 80 g for women. Level II BD corresponded to > 120 g but ≤ 180 g for men and > 80 g but ≤ 120 g for women. Level III BD was categorized as intake > 180 g for men and > 120 g for women (22, 39). Based on these thresholds, participants were classified into five drinking behavior groups: never drank, past drinker, non-BD, BD-I, and HIBD.

### 2.4 Statistical analysis

All statistical analyses were performed using Stata version 18.0. A two-sided *p*-value of < 0.05 was considered statistically significant. Continuous variables following a normal distribution were described as the mean ± standard deviation, and categorical variables were summarized as frequencies with percentages. Linear regression was used to assess the association between BD behavior or obesity and the TyG index. Logistic regression was applied to assess the associations between BD, the TyG index, and obesity and abnormal liver enzyme outcomes.

To visualize the overlap and co-occurrence of obesity, HIBD, and IR (as measured by the TyG index) in relation to liver enzyme abnormalities, a three-set Venn diagram was constructed. Participants were categorized based on the presence or absence of each risk factor. Given the lack of a universally accepted cutoff value for the TyG index, participants were stratified into tertiles (low, medium, and high) based on its distribution, with the highest tertile defined as the “high TyG” group for risk classification (40, 41). For each of the eight possible exposure combinations (no exposure, single, dual, or triple exposure), corresponding sample sizes and liver enzyme abnormality rates were calculated and graphically displayed.

**Figure 1:**
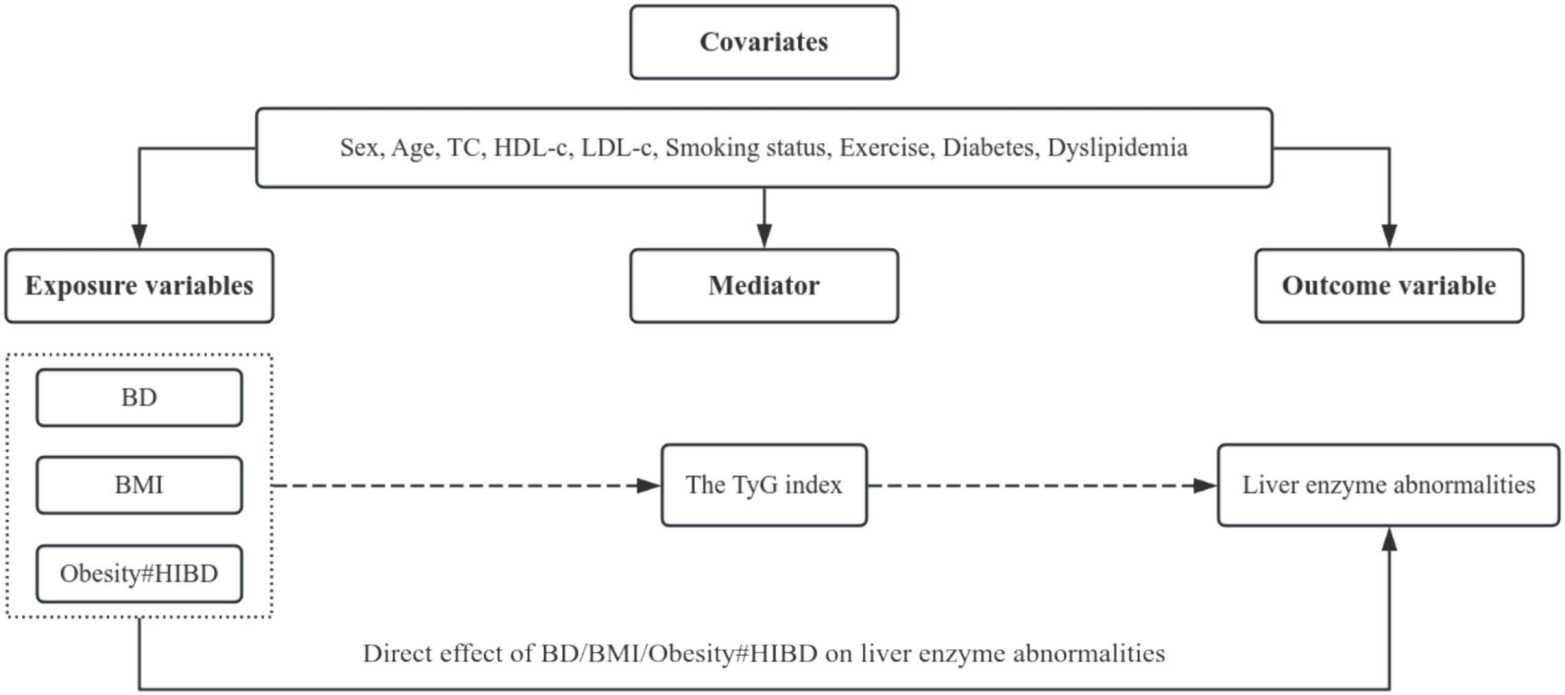
Hypothetical variable relationships diagram. BD, Binge drinking; BMI, Body mass index; HIBD, High-intensity binge drinking; Obesity#HIBD, Interaction term for obesity (BMI ≥ 25.00 kg/m^2^) and high-intensity binge drinking; TyG, Triglyceride–glucose; TC, Total cholesterol; HDL-c, High-density lipoprotein cholesterol; LDL-c, Low-density lipoprotein cholesterol. The diagram underlying this study illustrated the potential mechanisms through which the associations between BD levels, BMI, or the interaction term for obesity (BMI ≥ 25.00 kg/m^2^) and HIBD and liver enzyme abnormalities were mediated by the TyG index. All statistical models in this study were based on this structure and adjusted for sex, age, TC, HDL-c, LDL-c, smoking status, exercise, history of diabetes, and dyslipidemia. Dashed arrows represent indirect pathways mediated through the TyG index.

The hypothesized relationships among variables are illustrated in Figure 1. To investigate the mediating role of the TyG index in the associations between different BD intensities, obesity, their interaction, and liver enzyme abnormalities, causal mediation analysis was performed within a counterfactual framework (42–44). This approach decomposes the total effect (TE) of an exposure into two distinct components: the natural indirect effect (NIE), representing the portion of the effect mediated by the TyG index (i.e., the expected change in liver enzyme abnormalities when the TyG index changes due to the exposure), while holding the exposure constant, and the natural direct effect (NDE), representing the portion of the effect independent of the TyG index (44, 45) (i.e., the expected change in liver enzyme abnormalities if the exposure changed, while the TyG index is fixed at the level it would naturally take under the unexposed condition). For instance, a significant NIE implies that IR, represented by the TyG index, partially mediates the relationship between BD, obesity, or their interaction and liver enzyme abnormalities. A significant NDE indicates a direct effect of the exposure, independent of IR. To further evaluate the interaction between obesity (BMI ≥ 25.00 kg/m^2^) and HIBD, an interaction term was included in the mediation model, using the non-obese and non-binge-drinking group as the reference category. This approach allowed for the assessment of the extent to which IR mediates the joint impact of obesity and HIBD on liver enzyme abnormalities.

To ensure the robustness of the mediation effects, multiple sensitivity analyses were conducted. First, participants with baseline FBG levels ≥ 7 mmol/L were excluded (*n* = 5,324 in the full sample; *n* = 3,300 in the obese subgroup). Second, we excluded participants in the top 10% (full sample, *n* = 13,701) or 5% (obese subgroup, *n* = 4,500) of BMI values to address the influence of extreme body weight on outcomes. Third, underweight participants (BMI < 18.50 kg/m^2^, *n* = 2,938) were excluded to evaluate the impact of merging underweight and normal-weight individuals in the main analysis. Fourth, the potential impact of unmeasured confounding was examined. Finally, propensity score matching (PSM) was employed to balance baseline characteristics between groups, and mediation analyses were repeated in the matched sample. Additionally, subgroup analyses were conducted to explore whether the mediation effect of the TyG index varied by obesity status. Participants were stratified by BMI into non-obese (BMI < 25.00 kg/m^2^) and obese (BMI ≥ 25.00 kg/m^2^) groups.

## 3 Results

### 3.1 Basic characteristics of young and middle-aged participants

**Figure 2:**
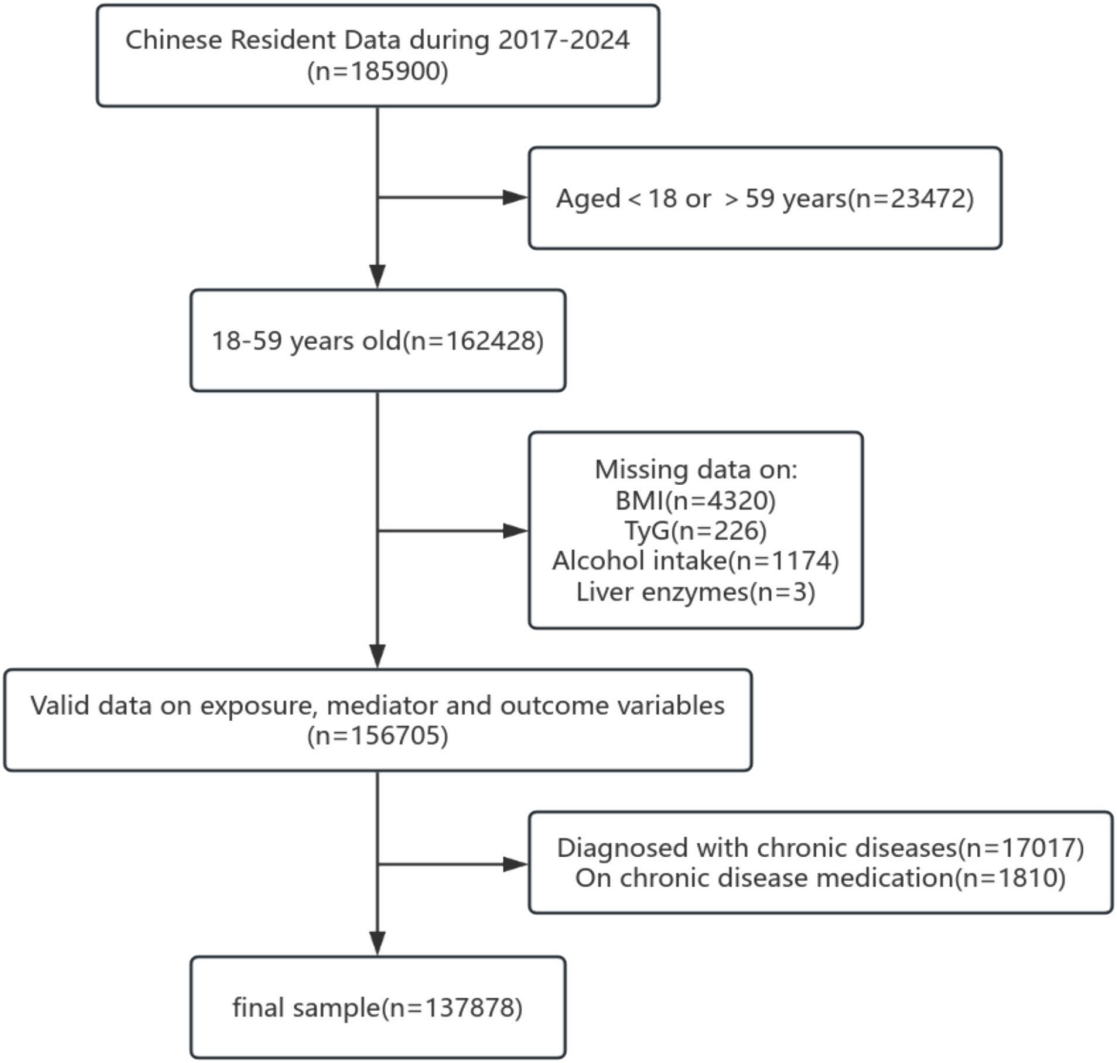
The flow chart of participant recruitment and selection process.

A total of 185,900 individuals were initially recruited for this study. After applying inclusion and exclusion criteria and removing cases with missing key variables, 48,022 participants were excluded. The final analytical sample included 137,878 participants, consisting of 71,453 men (51.8%) and 66,425 women (48.2%). The study flow is shown in Figure 2. The mean age of the participants was 40.98 ± 9.89 years. The overall prevalence of abnormal liver enzymes was 12.08%. Approximately one-third of the participants (45,852) were classified as obese. The prevalence of BD was 9.1%, with 1.5% of participants engaging in HIBD. The mean TyG index was 7.06. Participants with abnormal liver enzyme levels were younger and had lower HDL-c levels compared to those without abnormalities. In contrast, TC, TG, LDL-c, FBG, TyG index, obesity prevalence, and BD rates were all significantly higher in the abnormal liver enzymes group (*p* < 0.001; Table 1).

**Table 1:** Baseline characteristics and univariate analysis of all participants (*n* = 137,878).

Characteristic	Total (*n* = 137,878)	No abnormal liver enzymes (*n* = 121,227, 87.92%)	Abnormal liver enzymes (*n* = 16,651, 12.08%)	*χ*^2^/*t*^1^
Age (years)	40.98 ± 9.89	41.03 ± 9.92	40.62 ± 9.62	5.06^***^
Sex				2925.23^***^
Male	71,453 (51.80%)	59,554 (49.10%)	11,899 (71.50%)	
Female	66,425 (48.20%)	61,673 (50.90%)	4,752 (28.50%)	
TC (mmol/L)	5.00 ± 0.96	4.99 ± 0.95	5.09 ± 0.99	−12.69^***^
TG (mmol/L)	1.73 ± 1.75	1.69 ± 1.72	1.96 ± 1.89	−18.40^***^
HDL-c (mmol/L)	1.34 ± 0.30	1.34 ± 0.30	1.30 ± 0.30	18.67^***^
LDL-c (mmol/L)	2.89 ± 0.80	2.88 ± 0.79	2.92 ± 0.82	−4.82^***^
FBG (mmol/L)	5.40 ± 1.16	5.38 ± 1.13	5.53 ± 1.0.35	−16.11^***^
TyG index	7.06 ± 0.70	7.00 ± 0.67	7.49 ± 0.77	−86.64^***^
BMI categories				6057.19^***^
<23.00 kg/m^2^	59,719 (43.30%)	56,366 (46.50%)	3,353 (20.10%)	
23.00–24.99 kg/m^2^	32,307 (23.40%)	28,773 (23.70%)	3,534 (21.20%)	
≥25.00 kg/m^2^	45,852 (33.30%)	36,088 (29.80%)	9,764 (58.60%)	
BD intensity				1083.88^***^
Never	97,735 (70.90%)	87,553 (72.20%)	10,182 (61.10%)	
Stop	1,471 (1.10%)	1,237 (1.00%)	234 (1.40%)	
Non-BD	26,077 (18.90%)	22,224 (18.3%)	3,853 (23.1%)	
BD-I	10,471 (7.60%)	8,611 (7.10%)	1,860 (11.20%)	
HIBD	2,124 (1.50%)	1,602 (1.40%)	522 (3.1%)	
Diabetes Mellitus				2583.24^***^
No	119,640 (86.80%)	107,275 (88.50%)	12,365 (74.30%)	
Yes	18,238 (13.20%)	13,952 (11.50%)	4,286 (25.70%)	
Dyslipidemia				3593.39^***^
No	100,305 (72.70%)	91,421 (75.40%)	8,884 (53.40%)	
Yes	37,573 (27.30%)	29,806 (24.60%)	7,767 (46.60%)	
Smoking				978.49^***^
Never	98,291 (71.30%)	88,101 (72.70%)	10,190 (61.20%)	
Current	29,320 (21.30%)	24,377 (20.10%)	4,943 (29.70%)	
Past	3,884 (2.80%)	3,272 (2.70%)	612 (3.70%)	
Smoking				978.49^***^
Passive	6,383 (4.60%)	5,477 (4.50%)	906 (5.40%)	
Exercise				171.15^***^
No	53,789 (39.00%)	46,521 (38.40%)	7,268 (43.60%)	
Yes	84,089 (61.00%)	74,706 (61.60%)	9,383 (56.40%)	

*p < 0.05, **p < 0.01, ***p < 0.001. TC, Total cholesterol; TG, Triglycerides; HDL-c, High-density lipoprotein cholesterol; LDL-c, Low-density lipoprotein cholesterol; FBG, Fasting blood glucose; TyG, Triglyceride-glucose; BMI, Body mass index; BD, Binge drinking; BD-I, Level I binge drinking; HIBD, High-intensity binge drinking; ^1^p-values were calculated using the χ2 test for categorical variables and the t test for continuous variables.

### 3.2 Visualization of overlapping risk profiles and liver enzyme abnormalities

**Figure 3:**
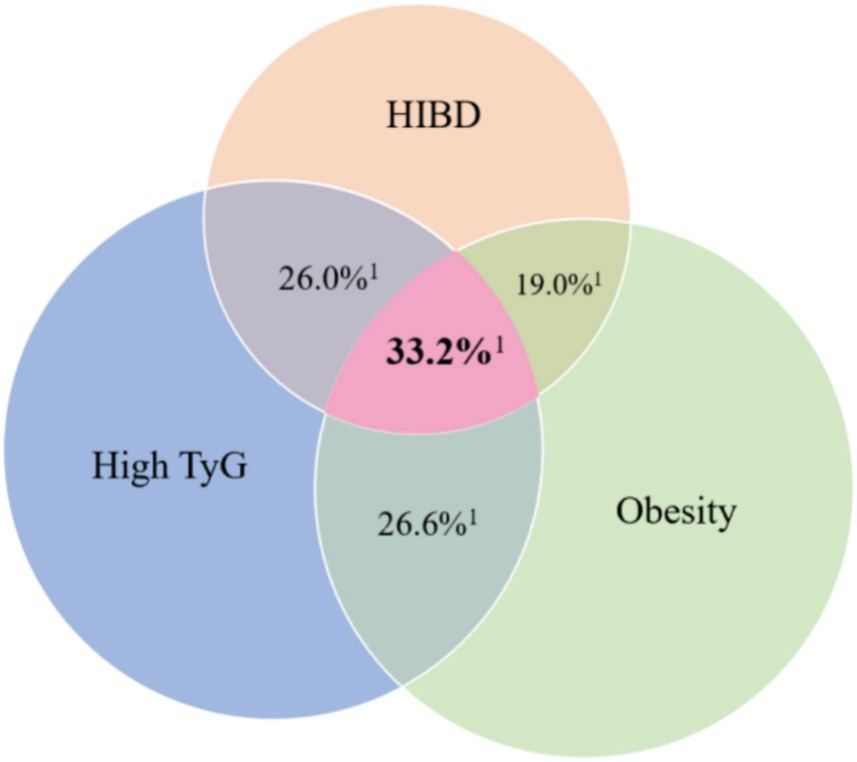
Venn diagram of high-intensity binge drinking, obesity, and insulin resistance showing corresponding liver enzyme abnormality rates. HIBD, High-intensity binge drinking; TyG, Triglyceride–glucose. ^1^Liver enzyme abnormality rates.

Figure 3 presents a Venn diagram illustrating the overlapping distribution of participants with HIBD, obesity, and IR, along with the corresponding liver enzyme abnormality rates in each exposure category. Detailed data are provided in Table 2. The highest abnormality rate (33.2%) was observed in participants exposed to all three risk factors (*n* = 996). In contrast, single-risk groups had lower rates. Participants without any of the three exposures showed the lowest abnormality rate (5.6%). These findings suggest a cumulative and potentially synergistic effect of the three factors on liver function.

**Table 2:** Liver enzyme abnormality rates by combinations of high-intensity binge drinking, obesity, and high TyG index.

Exposure combinations^1^	*N* (Participants)^2^	Liver enzyme abnormalities, *n* (%)
No risk factors	71,959	4,062 (5.6%)
Only HIBD	487	50 (10.3%)
Only Obesity	19,115	2,614 (13.7%)
Only High TyG	19,236	2,687 (14.0%)
HIBD + Obesity	332	63 (19.0%)
HIBD + High TyG	339	88 (26.0%)
Obesity + High TyG	25,420	6,764 (26.6%)
HIBD + Obesity + High TyG	996	321 (33.2%)

HIBD, High-intensity binge drinking; TyG, Triglyceride-glucose.

1 Exposure combinations are defined by the presence or absence of HIBD, obesity, and insulin resistance, defined as the highest tertile of the TyG index.

2 Total number of participants within each exposure combination.

### 3.3 Multivariable analysis

Multivariable analysis revealed that HIBD, obesity, and the TyG index were each independently associated with an increased risk of liver enzyme abnormalities. Participants with both obesity and HIBD had a 59.1% higher likelihood of liver enzyme abnormalities than those with neither risk factor (OR, 1.591; 95% CI, 1.401–1.806; Supplementary Table 1).

### 3.4 Mediation analysis

**Figure 4:**
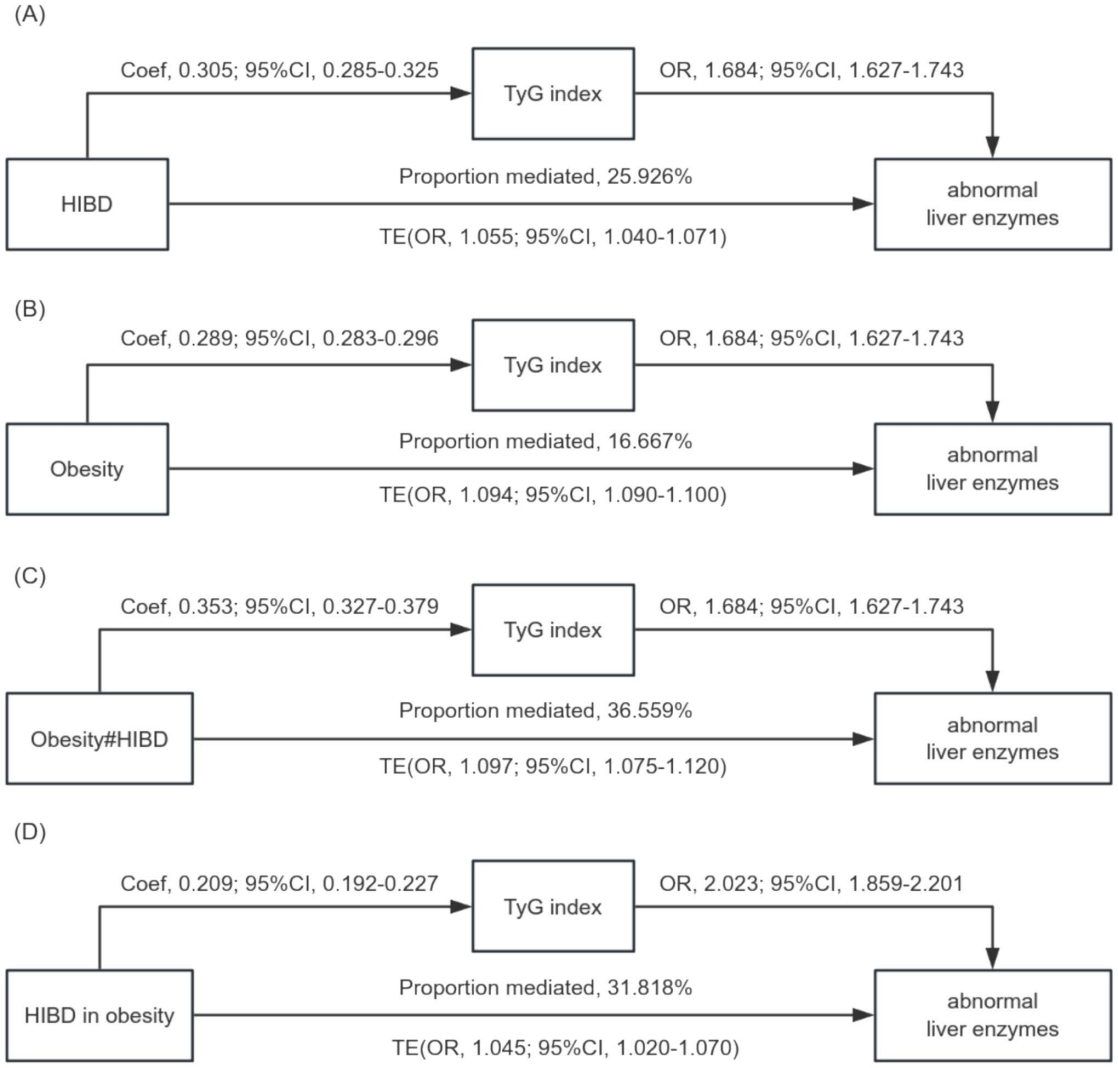
**(A)** Mediation role of TyG index in the relationship between high-intensity binge drinking and liver enzyme abnormalities. **(B)** Mediation role of TyG index in the relationship between obesity and liver enzyme abnormalities. **(C)** Mediation role of TyG index in the relationship between interaction term for obesity (BMI ≥ 25.00 kg/m^2^) and high-intensity binge drinking and liver enzyme abnormalities. **(D)** Mediation role of TyG index in the relationship between high-intensity binge drinking in obesity and liver enzyme abnormalities.

The decomposition of TE into NDE and NIE, mediated by the TyG index, is presented in Table 3 and visualized in Figure 4. Among individuals with obesity, the TyG index statistically explained 16.7% of the observed association with liver enzyme abnormalities (OR, 1.015; 95% CI, 1.075–1.083). For BD, a statistically significant indirect effect was observed only among participants in the HIBD group, where the TyG index accounted for 25.9% of the association (OR, 1.014; 95% CI, 1.012–1.016). The interaction between obesity and HIBD demonstrated the strongest indirect contribution, with the TyG index explaining 36.6% of the observed association (OR, 1.034; 95% CI, 1.029–1.039).

**Table 3:** Direct and TyG index-mediated associations of BMI categories, binge drinking levels, and the interaction between obesity (BMI ≥ 25.00 kg/m^2^) and high-intensity binge drinking with abnormal liver enzymes.

Exposure	TE OR(95% CI)	NDE OR(95% CI)	NIE OR(95% CI)	Proportion Mediated, %
BMI categories^1^^,^^2^^,^^4^^,^^5^				
<23.00 kg/m^2^	Reference	Reference	Reference	
23.00–24.99 kg/m^2^	0.985^***^(0.981,0.989)	0.984^***^(0.980,0.988)	1.001^***^(1.000,1.001)	−6.667
≥25.00 kg/m^2^	1.094^***^(1.090,1.100)	1.078^***^(1.075,1.083)	1.015^***^(1.075,1.083)	16.667
BD^1^^,^^2^^,^^4^^,^^5^				
Never	Reference	Reference	Reference	
Stop	1.003(0.988,1.018)	1.009(0.993,1.025)	0.994^***^(0.993,0.996)	--
Non-BD	0.998(0.994,1.003)	0.993^**^(0.989,0.997)	1.006^***^(1.005,1.006)	--
BD-I	1.006(1.000,1.012)	0.998(0.992,1.004)	1.008^***^(1.007,1.009)	--
HIBD	1.055^***^(1.040,1.071)	1.040^***^(1.027,1.055)	1.014^***^(1.012,1.016)	25.926
Obesity#HIBD^1^^,^^3^^,^^4^^,^^5^	1.097^***^(1.075,1.120)	1.061^***^(1.042,1.081)	1.034^***^(1.029,1.039)	36.559

*p < 0.05, **p < 0.01, ^***^p < 0.001. BMI, Body mass index; BD, Binge drinking; BD-I, Level I binge drinking; HIBD, High-intensity binge drinking; Obesity#HIBD, Interaction term for obesity (BMI ≥ 25.00 kg/m^2^) and high-intensity binge drinking; Coef, Coefficient; CI, Confidence interval; OR, Odds ratio; TE, Total effect; NDE, Natural direct effect; NIE, Natural indirect effect; Reference, Reference category.

1 All models were adjusted for sex, age, TC, HDL-c, LDL-c, smoking status, exercise, history of diabetes, and dyslipidemia.

2 When examining the mediating role of the TyG index between BMI categories and abnormal liver enzymes, binge drinking was included as a control variable. When examining the mediating role of the TyG index between binge drinking level and abnormal liver enzymes, BMI categories were included as a control variable.

3 The model used non-obese and non-high-intensity drinkers as the reference.

4 In the BMI categories model, the treatment-mediator interaction term was included, whereas in the binge drinking levels model and the obesity-high-intensity binge drinking interaction model, it was not included.

5 Causal mediation analysis was applied to decompose the total effect into natural indirect effect and natural direct effect; The mediate command in Stata 18.0 was used for analysis.

### 3.5 Subgroup analysis

Subgroup analysis stratified by BMI (non-obese group: BMI < 25 kg/m^2^; obese group: BMI ≥ 25 kg/m^2^) revealed distinct mediation effects of the TyG index in the association between HIBD and liver enzyme abnormalities (Table 4). In the obese group, the TyG index statistically accounted for 31.8% of the observed association between HIBD and liver enzyme abnormalities (OR, 1.014; 95% CI, 1.011–1.017), demonstrating a stronger indirect association compared to the non-obese group (16.9%; Figure 4).

**Table 4:** Subgroup analysis of the TyG Index mediation in the relationship between binge drinking levels and liver enzyme abnormalities, stratified by BMI.

Characteristic	BD Intensity	TE OR (95% CI)	NDE OR (95% CI)	NIE OR (95% CI)	Proportion Mediated, %
Non-obese (*n* = 92,026)^1^^,^^2^	Never (*n* = 71,776)	Reference	Reference	Reference	
	Stop (*n* = 817)	1.004(0.987,1.021)	1.008(0.990,1.026)	0.996^***^(0.994,0.998)	--
	Non-BD (*n* = 14,193)	0.997(0.993,1.002)	0.994^*^(0.989,0.999)	1.003^***^(1.003,1.004)	--
	BD-I (*n* = 4,414)	1.012^**^(1.004,1.020)	1.007(0.999,1.014)	1.005^***^(1.004,1.006)	--
	HIBD (*n* = 826)	1.065^***^(1.044,1.089)	1.055^***^(1.034,1.079)	1.011^***^(1.008,1.014)	16.923
Obese (*n* = 45,852)^1^^,^^2^	Never (*n* = 25,959)	Reference	Reference	Reference	
	Stop (654)	1.009(0.976,1.042)	1.015(0.982,1.049)	0.994^***^(0.990,0.997)	--
	Non-BD (*n* = 11,884)	1.009(1.000,1.019)	0.998(0.989,1.007)	1.011^***^(1.010,1.012)	--
	BD-I (*n* = 6,057)	1.017^**^(1.005,1.029)	1.004(0.992,1.016)	1.013^***^(1.012,1.015)	--
	HIBD (*n* = 1,298)	1.045^***^(1.020,1.070)	1.031^*^(1.007,1.055)	1.014^***^(1.011,1.017)	31.818

*p < 0.05, **p < 0.01, ***p < 0.001. BD, Binge drinking; BD-I, Level I binge drinking; HIBD, Heavy-intensity binge drinking; Coef, Coefficient; CI, Confidence interval; OR, Odds ratio; TE, Total effect; NDE, Natural direct effect; NIE, Natural indirect effect; Reference, Reference category.

1 All models were adjusted for sex, age, TC, HDL-c, LDL-c, smoking status, exercise, history of diabetes, and dyslipidemia.

2 Causal mediation analysis was applied to decompose the total effect into natural indirect effect and natural direct effect; The mediate command in Stata 18.0 was used for analyses.

### 3.6 Sensitivity analysis

Sensitivity analysis confirmed the robustness of the mediating role of the TyG index across all examined pathways, including HIBD, obesity, their interaction, and HIBD among individuals with obesity. Excluding participants with FBG ≥ 7 mmol/L slightly reduced the proportion of the association statistically explained by the TyG index for HIBD (from 25.9 to 24.5%) and for the interaction pathways (from 36.6 to 35.9%). Similar marginal decreases were observed when individuals in the top 10% of BMI were excluded. We also conducted a sensitivity analysis excluding underweight individuals, which yielded results consistent with the main analysis, supporting the methodological decision to merge underweight and normal-weight participants. Although unmeasured confounders led to moderate reductions in the proportions explained, these changes remained within an acceptable range. Following PSM, the proportion explained for HIBD decreased to 21.1%, while the effects observed in other pathways remained stable. Overall, these findings support the consistency and reliability of the observed indirect contributions of the TyG index across all sensitivity analyses (Supplementary Table 2).

## 4 Discussion

To the best of our knowledge, this is the first large-scale study in a young and middle-aged Chinese population to examine how IR, measured by the TyG index, statistically accounts for the associations between BD of varying intensities, obesity, their interaction, and liver enzyme abnormalities. While the overall prevalence of BD was 9.1%, it increased to 16.0% among individuals with obesity, highlighting a high-risk subgroup that warrants targeted investigation. Our findings show that IR statistically explained a meaningful proportion of the observed associations between HIBD, obesity, and liver enzyme abnormalities, with the greatest proportion observed for the interaction between HIBD and obesity. In contrast, no significant indirect contribution via IR was found for non-BD or low-intensity BD, suggesting an intensity-dependent metabolic mechanism. Sensitivity analysis validated the robustness of these results, and subgroup analysis revealed that the indirect role of IR was more pronounced in individuals with obesity. These results underscore the synergistic impact of obesity and HIBD on liver dysfunction through the pathway of IR. Although the effect sizes were modest, they were statistically significant and biologically meaningful, especially in high-risk populations such as individuals with obesity who engage in HIBD. These insights advance our understanding of the metabolic pathways linking lifestyle factors to liver injury and highlight the clinical relevance of targeting IR in prevention efforts.

Obesity and HIBD are two major risk factors for liver enzyme abnormalities, each contributing to liver injury through distinct but converging mechanisms. Obesity is characterized by a chronic low-grade inflammatory state, largely driven by VAT (46, 47). This state promotes the release of FFAs, which impair mitochondrial β-oxidation and increase the production of ROS, leading to oxidative stress and hepatocellular injury. FFAs also activate pro-inflammatory signaling pathways within hepatocytes, further aggravating liver damage (18). Similarly, HIBD exacerbates liver damage by increasing circulating lipopolysaccharides and pro-inflammatory cytokines, impairing mitochondrial function, and triggering oxidative stress and inflammatory responses, ultimately leading to hepatocyte apoptosis (12, 14). Additionally, HIBD worsens obesity-related metabolic disturbances. For instance, it accelerates the progression from simple steatosis to steatohepatitis and intensifies systemic and hepatic inflammation (13). When HIBD coexists with obesity, the combined metabolic burden leads to upregulation of hepatic cytochrome P450 2E1 (CYP2E1) activity and excessive ROS generation, exacerbating oxidative damage and liver dysfunction (9, 20). These findings suggest that obesity and HIBD not only have independent effects on liver injury but also interact synergistically through shared oxidative and inflammatory mechanisms, thereby increasing the likelihood and severity of liver enzyme abnormalities.

IR serves as a critical pathway linking HIBD and obesity with liver enzyme abnormalities, amplifying their synergistic effects. In obesity, elevated FFAs interfere with insulin signaling by impairing the tyrosine phosphorylation of IRS-1, which contributes to the development of IR, hepatic lipid accumulation, and oxidative stress (48). HIBD further contributes to hepatic inflammation and mitochondrial dysfunction, exacerbating IR through similar mechanisms (49). This convergence of metabolic disturbances exacerbates IR, which promotes hepatic lipid accumulation, increases CYP2E1 expression, and weakens antioxidant defenses, creating a feedback loop of oxidative stress and liver damage (27, 31, 32). Observational studies have linked excessive alcohol consumption (≥122 g/week) to an increased risk of type 2 diabetes (50), and liver enzymes such as ALT have been validated as a biomarker for IR (51). Together, these findings suggest that IR not only mediates the individual effects of obesity and HIBD but also serves as a shared mechanistic bridge through which these exposures jointly accelerate liver damage. Targeting IR may therefore represent a strategic intervention point for preventing or attenuating metabolically driven liver dysfunction.

Interestingly, no significant indirect contribution of IR was observed in individuals with non-BD or low-intensity BD, regardless of obesity status. Two explanations may account for this finding. First, the effects of BD on liver enzymes vary significantly by intensity (8). While HIBD increases the metabolic burden on the liver, moderate drinking may exert protective effects against hepatic steatosis (52, 53). In this study, moderate alcohol consumption appeared to act as a protective factor against liver enzyme abnormalities, and no significant association was found between low-intensity drinking and liver enzyme abnormalities. Second, low-intensity or moderate alcohol consumption is generally not linked to IR and may even improve insulin sensitivity (53). For example, consuming 1–2 glasses of wine per occasion has been shown to reduce oxidative stress, increase HDL-c, and elevate adiponectin levels (8). These effects may help mitigate metabolic dysfunction. Systematic reviews and observational studies have reported that moderate alcohol consumption enhances insulin sensitivity and reduces the risk of type 2 diabetes (54, 55). Potential mechanisms include increased levels of adiponectin and hepatic glutathione, both recognized as insulin sensitizers, as well as the modulation of inflammatory mediators and oxidative stress by ethanol and polyphenols in red wine (8, 54).

Building on the primary findings, subgroup analysis revealed that the indirect contribution of IR between HIBD and liver enzyme abnormalities was nearly twice as strong in individuals with obesity compared to those without. This finding suggests that the greater metabolic burden in individuals with obesity amplifies the adverse effects of HIBD on liver function via IR. These findings align with the primary results of this study, emphasizing IR as a key statistical mediator in the relationship between the interaction of obesity, HIBD, and liver enzyme abnormalities. Importantly, they underscore the synergistic nature of this interaction and highlight IR as a potential therapeutic target. Addressing IR in individuals with obesity may offer a focused strategy to mitigate alcohol-related liver damage more effectively than targeting either risk factor alone.

From a public health standpoint, our findings emphasize the need for targeted interventions in individuals with obesity who engage in HIBD, a group at particular high risk for liver dysfunction. Interventions that improve insulin sensitivity, such as structured lifestyle modifications (e.g., diet and exercise) or pharmacologic therapies, combined with efforts to reduce alcohol intake, may be especially effective in mitigating liver enzyme abnormalities in this population. Notably, this dual-targeted strategy may be more feasible and sustainable than interventions focused solely on weight reduction, which often face challenges in adherence and delayed therapeutic effects (56, 57). Given the central role of IR in statistically linking both obesity and HIBD to liver-related outcomes, IR represents a promising and actionable intervention target for reducing the burden of metabolically driven liver injury at the population level.

Several limitations were present in this study. First, its single-center cross-sectional design precludes causal inference. However, the use of causal mediation analysis was justified by strong theoretical and biological plausibility, supported by prior experimental evidence linking obesity and BD to IR and IR to liver injury. The counterfactual framework allows the decomposition of effects under assumed causal direction and minimal unmeasured confounding. While our findings offer insight into potential mediation pathways, they reflect statistical rather than causal mediation and should be interpreted accordingly. Second, liver function was assessed using only ALT and AST, omitting markers like gamma-glutamyl transferase and alkaline phosphatase, which may underestimate the true extent of liver injury. Third, IR was measured solely by the TyG index. The absence of homeostatic model assessment of IR and glycated hemoglobin A1c limited a more comprehensive metabolic assessment. Fourth, unmeasured factors such as genetic background and diet may confound the results, though sensitivity analyses suggest limited impact. Additionally, self-reported alcohol consumption may be influenced by cultural patterns specific to China. In certain regions, individuals frequently consume unrecorded, homemade alcoholic beverages with potentially high ethanol concentrations, which are often underreported (58). Moreover, alcohol use is commonly embedded in social occasions rather than solitary behavior (58), which may contribute to recall or social desirability bias. These factors could lead to an underestimation of BD intensity, particularly at higher levels.

In conclusion, this study identified IR as a critical mediator of the synergistic effect of obesity and HIBD on liver enzyme abnormalities. The pathways of liver injury differ by BD intensity, with minimal indirect contributions of IR observed in low-intensity drinking. These findings highlight the need for dual-target public health strategies, improving IR through lifestyle modification and implementing stepwise alcohol reduction plans. For high-risk individuals, such as individuals with obesity engaging in HIBD, early screening using the TyG index and targeted metabolic interventions may help prevent liver enzyme abnormalities and reduce the long-term burden of liver disease. Future research should adopt longitudinal designs, include diverse populations, and explore targeted interventions to enhance the understanding and management of these complex associations.

## Data Availability

The raw data supporting the conclusions of this article will be made available by the authors without undue reservation.
