# Supplementary material for: Insulin resistance as a mediator of the association between obesity, high-intensity binge drinking, and liver enzyme abnormalities in young and middle-aged adults: a cross-sectional study
Source: Front Nutr. 2025 Jul 24;12:1554392. doi: 10.3389/fnut.2025.1554392 (PMC12328162; doi:10.3389/fnut.2025.1554392)
Supplement: Supplementary file 1 [file Table_1.docx]

Supplementary Material

**Health Self-assessment Questionnaire(Key Excerpts)**

I Basic information

1 Sex： □Male □Female

2 Age： years old

II Current medical history

1 Do you have a clearly diagnosed disease or abnormality?

A.Yes B.No

1-1 Please identify the name of the specific disease or abnormality: (Multiple choices allowed)

A. Hypertension B. Stroke C. Coronary Heart Disease

D. Peripheral Vascular Disease E. Diabetes F. Fatty Liver Disease

G. Chronic Kidney Disease H. Chronic Gastritis or Gastric Ulcer

I. Helicobacter pylori Infection J. Gastric Polyps K. Intestinal Polyps

L. Chronic Obstructive Pulmonary Disease (COPD) M. Asthma

N. Chronic Pancreatitis O. Osteoporosis P. Chronic Hepatitis or Liver Cirrhosis

Q. Chronic Cholecystitis or Gallstones R. Tuberculosis S. Rheumatoid Arthritis

T. Prostatitis or Prostate Enlargement U. Chronic Breast Disease

V. Human Papillomavirus (HPV) Infection W. Dyslipidemia

X. Elevated Uric Acid Y. Malignant Tumors Z. Others

1-2 Please specify the type of malignant tumor you have been diagnosed with:

A. Lung Cancer B. Liver Cancer C. Gastric Cancer E. Esophageal Cancer

F. Colorectal Cancer G. Leukemia H. Brain Tumor I. Breast Cancer

J. Pancreatic Cancer K. Bone Cancer L. Bladder Cancer

M. Nasopharyngeal Cancer N. Cervical Cancer O. Uterine Cancer

P. Prostate Cancer Q. Ovarian Cancer R. Thyroid Cancer

S. Skin Cancer T. Others

1-3 Please indicate the age at which you were diagnosed with the above disease or condition: ______ years old

III Medication history

1 Have you been taking medications long-term? (Defined as continuous use for over 6 months, at least once daily on average)

A.Yes B.No

1-1 Which medications have you been taking long-term? (Multiple choices allowed)

A. Antihypertensive drugs B. Antidiabetic drugs C. Lipid-lowering drugs

D. Uric acid-lowering drugs E. Antiarrhythmic drugs F. Asthma-relief medications

G. Antipyretic and analgesic drugs H. Prednisone or similar drugs

I. Estrogen medications J. Diuretics K. Sedatives or sleeping pills

L. Traditional Chinese medicine M. Contraceptive pills N. Antidepressants

O. Others

IV Lifestyle Habits

1 Do you smoke? (Defined as smoking continuously for more than one year)

A. Do not smoke B. Smoke C. Smoked before, but quit (quit smoking for over one year) D. Passive smoking (exposure for more than 15 minutes per day and at least one day per week)

1-1 How many cigarettes do you usually smoke per day? (Include before quitting)

_____ cigarettes; How many years have you been smoking continuously? (Include before quitting) _____ years

1-2 How long have you been smoke-free? _____ years

2 Do you drink alcohol? (Defined as drinking at least once a week on average)

1. Do not drink B. Drink

C. Drank before, but quit (quit drinking for over one year)

2-1 What type of alcohol do you usually consume?

A. Chinese liquor B. Beer C. Wine D. Drink all types

2-2 How many times per week do you drink? (Include before quitting)

A. 1–2 times B. 3–5 times C. More than 5 times

2-3 How much do you drink each time? (1 unit equals 50ml of spirits, 100ml of wine, or 300ml of beer)

A. 1–2 units B. 3–4 units C. More than 5 units

2-4 How many years have you been drinking continuously? (Include before quitting) _____ years

2-5. How long have you been alcohol-free? _____ years

3 Do you participate in physical exercise?

A. Do not participate

B. Occasionally participate

C. Regularly participate (defined as exercising at least 3 times per week for more than 30 minutes per session)

HDL-c, LDL-c were measured using enzymatic methods on an automated biochemical analyzer. Diabetes was defined based on medical history, FBG (≥7.0 mmol/L), or hemoglobin A1c (HbA1c ≥6.5%) results[1]. Dyslipidemia was defined based on medical history or if TC ≥ 6.2 mmol/L, TG ≥ 2.3 mmol/L, LDL-c ≥ 4.1 mmol/L, or HDL-c < 1.0 mmol/L[2].

**Supplementary Table 1.** Multivariable analysis of the relationship between abnormal liver enzymes and binge drinking, BMI categories, interaction term for obesity (BMI ≥ 25.00 kg/m²) and high-intensity binge drinking and TyG index.

| Exposure | Abnormal Liver Enzymes |
| --- | --- |
|  | OR(95%CI) |
| BD^1^ |  |
| Never | Reference |
| Stop | 1.073(0.924,1.247) |
| Non-BD | 0.947^*^(0.901,0.995) |
| BD-I | 0.971(0.910,1.035) |
| HIBD | 1.353^***^(1.212,1.512) |
| BMI categories^1^ |  |
| ＜23.00 kg/m^2^ | Reference |
| 23.00-24.99kg/m^2^ | 1.581^***^(1.500,1.665) |
| Exposure | Abnormal Liver Enzymes |
|  | OR(95%CI) |
| BMI categories^1^ |  |
| ≥25.00kg/m^2^ | 2.662^***^(2.537,2.793) |
| Obesity#HIBD^1,2^ | 1.591^***^(1.401,1.806) |
| TyG index^1^ | 1.684^***^(1.627,1.743) |

**p*＜0.05, ***p*＜0.01, ****p*＜0.001

Abbreviations: BD, Binge drinking; BD-I, Level I binge drinking; HIBD, High-intensity binge drinking; BMI, Body mass index; Obesity#HIBD, Interaction term for obesity (BMI ≥ 25.00 kg/m²) and high-intensity binge drinking; TyG, Triglyceride-glucose; OR, Odds ratio; CI, Confidence interval; Reference, Reference category.

^1^ All models were adjusted for sex, age, TC, HDL-c, LDL-c, smoking status, exercise, history of diabetes, dyslipidemia.

^2^ The reference category for the interaction between obesity and high-intensity binge drinking is non-obese and non-high-intensity drinkers.

**Supplementary Table 2.** Sensitivity analysis of the mediating role of the TyG index across different pathways in relation to liver enzymes abnormalities.

| **Exposure** | **TE**  **OR(95% CI)** | **NDE(DE)**  **OR(95% CI)** | **NIE(ACME)**  **OR(95% CI)** | **Proportion mediated, %**  **(% of Tot Eff mediated)** |
| --- | --- | --- | --- | --- |
| Sensitivity analysis 1: Excluding participants with baseline FBG≥7 mmol/L^1,2,3,5^ | | | | |
| HIBD(All) | 1.054^***^(1.039,1.070) | 1.041^***^(1.026,1.055) | 1.013^***^(1.011,1.015) | 24.528 |
| HIBD(In obesity) | 1.043^**^(1.016,1.070) | 1.028^*^(1.002,1.057) | 1.014^***^(1.009,1.018) | 33.333 |
| Obesity | 1.092^***^(1.088,1.096) | 1.075^***^(1.071,1.080) | 1.015^***^(1.014,1.016) | 17.045 |
| Obesity#HIBD | 1.096^***^(1.074,1.120) | 1.060^***^(1.041,1.081) | 1.034^***^(1.029,1.038) | 35.870 |
| Sensitivity analysis 2: Excluding participants in the top 10% of BMI values ^1,2,3,5^ | | | | |
| HIBD(All) | 1.059^***^(1.042,1.077) | 1.046^***^(1.030,1.063) | 1.012^***^(1.010,1.014) | 21.053 |
| HIBD(In obesity) | 1.041^**^(1.016,1.067) | 1.029^*^(1.006,1.054) | 1.011^***^(1.009,1.014) | 27.500 |
| Obesity | 1.063^***^(1.058,1.067) | 1.051^***^(1.046,1.055) | 1.011^***^(1.010,1.012) | 18.033 |
| Obesity#HIBD | 1.090^***^(1.062,1.117) | 1.059^***^(1.035,1.083) | 1.028^***^(1.024,1.034) | 32.558 |
| Sensitivity analysis 3: Excluding participants with underweight ^1,2,3,5^ | | | | |
| HIBD(All) | 1.056^***^(1.040,1.071) | 1.039^***^(1.025,1.054) | 1.016^***^(1.014,1.018) | 29.630 |
| Obesity | 1.099^***^(1.094,1.104) | 1.080^***^(1.076,1.085) | 1.017^***^(1.016,1.018) | 18.085 |
| **Exposure** | **TE**  **OR(95% CI)** | **NDE(DE)**  **OR(95% CI)** | **NIE(ACME)**  **OR(95% CI)** | **Proportion mediated, %**  **(% of Tot Eff mediated)** |
| Sensitivity analysis 3: Excluding participants with underweight ^1,2,3,5^ | | | | |
| Obesity#HIBD | 1.091^***^(1.069,1.113) | 1.062^***^(1.043,1.082) | 1.027^***^(1.024,1.030) | 31.034 |
| Sensitivity analysis 4: Accounting for the potential presence of unobserved confounders ^1,2,3,5^ | | | | |
| HIBD(All) | 1.074(1.061,1.089) | 1.060(1.047,1.074) | 1.013(1.012,1.015) | 18.478(0.154,0.222) |
| HIBD(In obesity) | 1.049(1.026,1.074) | 1.037(1.013,1.060) | 1.012(1.010,1.015) | 25.568(0.175,0.486) |
| Obesity | 1.100(1.097,1.105) | 1.085(1.081,1.090) | 1.014(1.013,1.015) | 14.931(0.143,0.155) |
| Obesity#HIBD | 1.124(1.106,1.143) | 1.093(1.076,1.112) | 1.029(1.026,1.031) | 24.243(0.213,0.282) |
| Sensitivity analysis 5: Using propensity score matching ^1,2,3,4,5^ | | | | |
| HIBD(All) | 1.059^***^(1.042,1.075) | 1.046^***^(1.029,1.063) | 1.012^***^(1.008,1.015) | 21.053 |
| HIBD(In obesity) | 1.044^***^(1.020,1.069) | 1.031^**^(1.008,1.055) | 1.013^***^(1.010,1.015) | 30.233 |
| Obesity | 1.093^***^(1.089,1.099) | 1.076^***^(1.071,1.080) | 1.016^***^(1.015,1.017) | 17.978 |
| Obesity#HIBD | 1.097^***^(1.075,1.120) | 1.061^***^(1.042,1.081) | 1.034^***^(1.029,1.038) | 36.559 |

Abbreviations: TyG, Triglyceride-glucose; FBG, Fasting blood glucose; BMI, Body mass index; BD-I, Level I binge drinking; HIBD, High-intensity binge drinking; Obesity#HIBD, Interaction term for obesity (BMI ≥ 25.00 kg/m²) and high-intensity binge drinking; Coef, Coefficient; CI, Confidence interval; OR, Odds ratio; TE ,Total effect; NDE, Natural direct effect; NIE, Natural indirect effect; Reference, Reference category.

^1^ All models were adjusted for sex, age, TC, HDL-c, LDL-c, smoking status, exercise, history of diabetes, dyslipidemia.

^2^ When examining the mediating role of TyG index between obesity and abnormal liver enzymes, HIBD was included as a control variable. When examining the mediating role of TyG index between HIBD and abnormal liver enzymes, BMI categories were included as a control variable.

^3^ The model used BMI <23.00 kg/m², never drinker or non-obese and non-high-intensity drinkers as the reference. In obesity model, the treatment-mediator interaction term was included, whereas in HIBD model and the obesity-HIBD interaction model, it was not included.

^4^ PSM was conducted using a logistic regression model to estimate propensity scores based on covariates. Matching was performed using the nearest-neighbor method with 1:1 matching (neighbor(1)) and a caliper width of 0.05 (caliper(0.05)), without replacement. The quality of matching was evaluated by ensuring standardized mean differences (SMDs) for all covariates were <0.1.

^5^ Causal mediation analysis was applied to decompose the total effect into natural indirect effect and natural direct effect; The mediate command in Stata 18.0 was used for analysis.

**References**

1. Harreiter J, Roden M. [Diabetes mellitus: definition, classification, diagnosis, screening and prevention (Update 2023)]. Wien Klin Wochenschr (2023)135:7-17.doi:10.1007/s00508-022-02122-y

2. Tian X, Chen S, Wang P, Xu Q, Zhang Y, Luo Y, et al. Insulin resistance mediates obesity-related risk of cardiovascular disease: a prospective cohort study. Cardiovasc Diabetol (2022)21:289.doi:10.1186/s12933-022-01729-9
